# Twentieth Century Practice

**Published:** 1897-10

**Authors:** 


TWENTIETH CENTURY PRACTICE. An International Encyclopedia of Modern Medical Science. By Leading Authorities of Europe and America. Edited by Thomas L. Stedman, M. D., New York City. In Twenty Volumes. Volume XI. "Diseases of the Nervous System." New York: William Wood & Company. 1897.

Dr. James Hendrie Lloyd, of Philadelphia, contributes the opening chapter in this volume on "Diseases of the Cerebro-Spinal and Sympathetic Nerves." This is an extensive article, covering 476 pages, and embraces the following special subjects: General anatomy of the nerves; general physiology of the nerves; electrotonus; modes of sensation; the reflexes; general pathology; general symptomatology; disorders of motion; disorders of sensation; disorders of nutrition; disorders of the reflexes; disorders of the circulation; diseases of the cranial nerves, including combined palsies of the ocular muscles, headache, tic douloureux, torticollis, etc.; diseases of the spinal nerves, including sciatica, multiple neuritis and diseases of the cauda equina; diseases of the sympathetic nervous system.

The second contribution is by Dr. Charles K. Mills, of Philadelphia, on "Trophoneuroses" (excluding scleroderma, acromegaly, and adiposis dolorosa).

The following are the special subjects treated of in this chapter: Hemifacial atrophy; hemilingual atrophy; hemifacial hypertrophy; hypertrophy of one-half of the body; locilized atrophies and hypertrophies, hyperostosis of the cranium; Raynaud's disease; perforating ulcer of the foot; ainhum.

Dr. F. X. Dercum, of Philadelphia, writes on scleroderma, acromegaly, and adiposis dolorosa.

"Diseases of the Spinal Cord," is the subject of an article by Drs. L. Bruns, of Hanover, and F. Windscheid, of Leipsic. In this chapter the following diseases are treated of: Injuries of the spinal cord; spinal caries; arthritis deformans of the spine; lateral curvature of the spine; tumors of the spinal cord; tumors of the spinal column; tumors of the spinal meninges and cord; Hæmatomyelia; pachymeningitis cervicalis hypertrophica; acute leptomeningitis; chronic leptomeningitis; poliomyelitis anterior acuta infantum; poliomyelitis anterior acuta adultorum; poliomyelitis anterior subacuta; poliomyelitis anterior chronica; myelitis; abscess of the spinal cord; syphilis of the spinal cord; syringomyelia; spastic spinal paralysis; progressive spinal muscular atrophy. Dr. P. J. Mobius, of Leipsic, contributes a very interesting article on "Tabes Dorsalis," and Dr. Adolf Strumpel, of Erlangen, one on the "Combined System Diseases of the Spinal Cord," including hereditary ataxia (Friedreich's Disease); hereditary spastic spinal paralysis; and secondary system disease. The closing chapter is by Dr. Lightner Witmer, of Philadelphia, on "Pain."

The foregoing list of subjects, and the eminent medical men who contributed the different articles, will give some idea of the scope and importance of this volume. These are all exhaustive contributions, written by men who are fully abreast of the times; the volume is, therefore, one of the best on these subjects, and may be considered authoritative. S. E. H.

